# THE COMPARISON OF INTELLIGENCE QUOTIENTS OF ATOPIC AND NONATOPIC CHILDREN IN IBADAN, NIGERIA

**DOI:** 10.4103/0019-5154.70665

**Published:** 2010

**Authors:** O O M Daramola, O O Ayoola, A O Ogunbiyi

**Affiliations:** *From the Dermatology Division, Department of Medicine, University of Ilorin, Ilorin, Nigeria*; 1 *From the Department of Paediatrics, College of Medicine, University of Ibadan, Ibadan, Nigeria*; 2 *From the Department of Medicine, College of Medicine, University of Ibadan, Ibadan, Nigeria*

**Keywords:** *Atopy*, *intelligence quotient*, *atopic dermatitis*

## Abstract

**Background:**

Atopy-related illnesses such as atopic dermatitis and asthma are chronic illnesses, and children suffering from such illnesses are subjected to frequent absenteeism from school. Studies have shown that the performance of children with asthma was comparable to their healthy counterparts despite their absenteeism at school, in contrast to findings in other chronic illnesses like epilepsy.

**Aim:**

In the present study, we investigated the association between atopy and intelligence quotient (IQ) scores in a group of Nigerian children in Ibadan, a city in southwestern Nigeria.

**Materials and Methods:**

This is a cross-sectional study of children in an urban elementary school. Questionnaires to ascertain the presence of atopy-associated conditions such as hay fever, atopic dermatitis, asthma, allergic rhinitis, and allergic conjunctivitis were administered to the parents of 128 pupils in the 3^rd^ to 6^th^ grades of elementary school. Based on the responses to the questionnaire, pupils were categorized as being atopic and nonatopic. All the pupils underwent the Standard Progressive Matrices IQ test. The IQ scores were then compared among these two groups of children.

**Results:**

Out of the children studied, 26.6% were found to have atopy and after adjusting for factors such as age and sex, the IQ scores in this atopic group were not found to be statistically different from the scores in the nonatopic group (r = 2.122872, *P* = 0.009).

**Conclusion:**

IQ scores were not statistically significantly different for children with and without atopy. Thus, the presence of atopy does not appear to be associated with low IQ scores and hence, may not be related to poor school performance.

## Introduction

The definition of intelligence has generated intense controversy amongst psychologists with no consensus to date.[1] Various definitions exist for intelligence, including i) the capacity of abstract thinking and reasoning,[1] ii) the ability that allows learning and amassing knowledge,[2] and iii) the ability to solve problems posed by Nature.[2] However, in spite of these varying schools of thoughts among psychologists, many agree that there is an interplay of factors like cognitive speed, motivation, adjustment, heredity, and the environment.[3,4]

The effect of chronic illnesses on cognitive function in childhood has been the interest of many researchers. The effects of chronic illnesses such as epilepsy,[5] childhood cancer,[6] HIV,[7] diabetes mellitus[8] and asthma[9] on cognition have been widely studied and reported. Of all these chronic illnesses, it is interesting that extrinsic asthma usually associated with atopy, has not been found to seriously impair cognition in childhood unlike other chronic illnesses,[9] and their performance is comparable with that of their healthy counterparts despite the time lost in school due to absenteeism and their relatively ill health.[10]

The question then arises as to whether there is any difference in the intelligence quotients of atopic children compared to their nonatopic counterparts. Therefore, we sought to evaluate and compare intelligence quotients of children with atopic diathesis and those without atopic diathesis using the Standard Progressive Matrices test that has been tested and validated among Nigerian children.

## Materials and Methods

Our study included 128 healthy children from an urban private school in Ibadan, a city in southwestern Nigeria with both urban and semi-urban areas. The study site, Agodi, which was randomly selected from other urban areas, had five private nursery and primary schools from which the school under study was selected.

The study was commenced after permission was obtained from the school authorities and all children in their 3^rd^ to 6^th^ years of elementary school were recruited into the study after informed consent was obtained from them and their parents. A questionnaire was administered to the parents of the children under study to determine the presence of atopy All the children underwent an intelligence test (progressive matrices intelligence test) to assess their intelligence quotients.

Instrument: The standard progressive matrices test was used to assess the intelligence of all the subjects; this is a nonverbal test of an individual’s intellectual capacity. Therefore, an individual is not penalized for his/her inability to read or understand the English language. The scale consists of 60 matrix items divided into five sets of 12 items each. Each set consists of matrices of increasing difficulty. While the earlier series require accuracy of visual discrimination, the later ones involve two-dimensional analogies which demand permutation, alteration of pattern, and perception of other logical relations for successful solution. Evidence for the reliability and validity of Progressive Matrices in Nigeria already exists; the reliability level is 0.70–0.90.

Data Analysis: All data were analyzed using the SPSS software package for Windows. The presence or absence of atopy in the children was determined and these children were assigned to two groups: Group 1 with atopy, group 2 without atopy. The IQ scores obtained from the progressive matrices test were grouped into five categories in ascending order for the purpose of comparison. The IQ scores in the two groups were then compared. The means of continuous variables were compared using Student’s t-test or analysis of variance (ANOVA). Categorical data were compared using the Chi-square test. Multinomial logistic regression was also done for all the variables to identify the relationship between categorized IQ scores and atopy. Differences were deemed to be statistically significant where *P* < 0.05.

## Results

A total of 128 children were enrolled into the study, comprising of 66 (51.6%) boys and 62 (48.4%) girls with a male to female ratio of 1: 0.9. The children were in the 3^rd^ to 6^th^ grades of elementary school (referred to as primary 3 to 6 in this environment) and their ages ranged from 6 to 12 years with a mean ± SD age of 9 ±1 years. Forty-one children were in primary 3, 42 in primary 4, 33 in primary 5, and 12 in primary 6.

Thirty-four (26.6%) children had atopy and the remaining 94 (73.4%) had no atopy. The mean IQ score of children with atopy was 24.5 ±11.2 whereas the mean score of children without atopy was 23.2 ±11.8. There was no statistically significant difference in the mean scores of the two groups of children (t = -0.579, *P* = 0.57).

The IQ scores were grouped into five categories based on the comparison scale for scores on the progressive matrices (Appendix 1). There was no statistically significant difference in the IQ score categories of the children with positive history of atopy compared with those with no history of atopy (χ^2^ = 7.719, *P* = –0.102) as shown in Table 1.

**Table 1:** Categorization of IQ score based on the presence of atopy

Atopy	IQ score category				
	1 (%)	2 (%)	3 (%)	4 (%)	5 (%)
Present (*n* = 34)	6 (17.6)	11 (32.4)	10 (29.4)	6 (17.7)	1 (2.9)
Absent (*n* = 94)	4 (4.3)	46 (48.9)	29 (30.9)	12 (12.8)	3 (3.2)

χ^2^ = 0.136, *P* = 0.712

There was no significant difference between mean ±SD IQ scores of boys and girls, 25.0 ±11.5 and 22.0 ±11.5 respectively (t = 1.469, *P* = 0.144). As shown in Table 2, there was no statistically significant difference between the IQ score categories and sex (χ^2^ = 1.794, *P* = 0.774). However, IQ categories were different in each grade with most of the children (73.2 and 42.9% respectively) in lower grades, namely, primary 3 and 4, having a lower IQ score category of 2.

**Table 2:** Categorization of IQ score based on gender and school grade

Parameter	IQ score category					
	1.00	2.00	3.00	4.00	5.00	Total
Sex						
Boy	5 (7.6)	26 (39.4)	23 (34.9)	10 (15.2)	2 (3.0)	66 (100)
Girl	5 (8.1)	31 (50)	16 (25.8)	8 (12.9)	2 (3.2)	62 (100)
Total	10 (7.8)	57 (44.5)	39 (30.5)	18 (14.1)	4 (3.1)	128 (100)
χ^2^ = 1.7940 *P* = 0.774						
Class						
3	1 (2.4)	30 (73.2)	6 (14.6)	3 (7.3)	1 (2.4)	41 (100)
4	5 (11.9)	18 (42.9)	12 (28.6)	6 (14.3)	1 (2.4)	42 (100)
5	4 (12.1)	8 (24.2)	16 (48.5)	4 (12.1)	1 (3.0)	33 (100)
6	0 (0.0)	1 (8.3)	5 (41.7)	5 (41.7)	1 (8.3)	12 (100)
Total	10 (7.8)	57 (44.5)	39 (30.5)	18 (14.1)	4 (3.1)	128 (100)
χ^2^ = 34.8274, *P* = 0.0001						

Further analysis comparing IQ score and atopy with grade as the stratifying factor shows a higher IQ score category among nonatopic children only in primary 4 (χ^2^ = 17.772, *P* = 0.001). Scores were similar in atopic and nonatopic children in all other grades [Table 3].

**Table 3:** Association of IQ category with atopy stratified by grade

Parameter		IQ Category					Atopy	
		1	2	3	4	5	χ^2^	*P* value
Class								
3	Yes	1 (8.3)	6 (50)	3 (25)	1 (8.3)	1 (8.3)	6.513	0.164
	No	0 (0)	23 (79.3)	4 (13.8)	2 (6.9)	0 (0)
4	Yes	5 (41.7)	2 (16.7)	2 (16.7)	3 (25)	0 (0)	17.772	*0.001
	No	0 (0)	16 (53.3)	10 (33.3)	3 (10)	1 (3.3)		
5	Yes	0 (0)	2 (28.6)	4 (57.1)	1 (14.3)	1 (3.8)	1.587	0.811
	No	4 (15.4)	6 (23.1)	12 (46.2)	3 (11.5)	0 (0)		
6	Yes	-	1 (33.3)	1 (33.3)	1 (33.3)	0 (0)	3.467	0.325
	No	-	0 (0)	4 (44.4)	4 (44.4)	1 (11.1)		

* *P* < 0.05

However, the presence of atopy was associated with lower IQ class 2 when all other factors such as age, sex, and grade were controlled for in multinomial logistic regression [Table 4].

**Table 4:** Association between IQ, grade and Atopy on multinomial logistic regression

IQ Class	β	SE(β)	*P* value
1 (Reference)			
2	-1.836	0.728	0.012*
3	-1.470	0.742	0.084
4	-1.099	0.816	0.178
5	-1.504	1.323	0.256

Pseudo R^2^ =0.021,

* *P* < 0.05, Adjusted for age, sex, and grade

## Discussion

There is a paucity of literature on the relationship between atopy and intelligence in children. It may more appropriate to state that we found no such documentation at the time of this study. However, literature does contain study reports on the effect of chronic illness on cognitive function in children. Studies have shown that chronic illnesses such as epilepsy[5] tend to adversely affect cognition in children whereas extrinsic asthma does not adversely affect cognition in children.[9] Asthmatic children fared comparably with their nonasthmatic counterparts in spite of their absenteeism from school.[9] Asthma in children is associated with atopy, which is also considered to be present in an individual with hay fever, recurrent allergic conjunctivitis, and atopic dermatitis. In 70% of these individuals, there is a family history of atopy with one or more of the atopy-related illnesses occurring in first-degree family members. This concept of genetic susceptibility is being strengthened by the identification of a predisposing gene on chromosome 11q13 in patients with asthma.[13] Although no gene has been identified for atopic dermatitis, an autosomal dominant pattern of inheritance has been suggested.[14]

Atopy was present in 26.6% of the pupils in this study. The prevalence of atopy among Nigerians has not been documented; however, a previous study on atopic dermatitis from Nigeria reported that atopic dermatitis accounted for 3–5% of dermatology consultations.[15] A more recent study from Ibadan reported prevalence of atopic dermatitis among children attending the skin clinic to be 5.87%.[16] This increase is in conformity with the global trend in which the prevalence of atopy has been on the increase. This is believed to be the effect of increased urban industrialization and the resultant environmental pollution.[17] Recently, the use of antibiotics and antipyretics in the first year of life has been thought to contribute to this global increase in the prevalence of atopy.[14] Increased urban industrialization, environmental pollution, use of antibiotics early in life, and a decrease in infections and infestations may lead to an increase in the expression of atopy in those genetically predisposed who may not have expressed it if these environmental changes had not occurred.

The need to have an IQ test in conformity with the sociocultural environment was addressed by employing the progressive matrices test that was designed by Nigerian psychologists for Nigerian school children and validated among Nigerian pupils.

The fact that the IQ score increased in the higher grades was not unexpected as brain development and abstract information are expected to increase with increasing age and time in school. There was no gender difference in the IQ scores among pupils in the same grade.

In this study, only atopic children in primary 4 had lower IQ scores than nonatopic children. However, when we controlled for age and gender, lower IQ scores was associated with atopy. It is of note that there are limitations in using IQ scores as a measure of intelligence because it has been found that some individuals who had low scores in IQ tests did well while performing some other tasks. In spite of this limitation, the IQ test provides an objective way of assessing intelligence.

The finding of association between atopy and lower IQscore when age and gender were corrected for is an indication that there could be some effect of the consequences of frequent absence from school and other factors related to atopic conditions on these children such as victimization.

As atopy-related illnesses such as atopic dermatitis are chronic illnesses, affected children are subjected to frequent absenteeism from school and they may hardly attain their full potential for learning. In addition, they could be ostracized, leading to untold psychological trauma and some of these children eventually becoming school dropouts. It is therefore imperative that if this unfortunate trend must be stopped with continuous public enlightenment programs and health education of parents and teachers.

## Conclusion

Although the subject of intelligence and the factors affecting it are still controversial, there is the need for follow-up studies to assess the long term effects of atopyrelated conditions in childhood.

## Recommendations

Further studies are needed to ascertain the public attitude towards children with atopy-related diseases such as atopic dermatitis, and their effects on children.

## Appendix 1

**Table:** Rough comparison scale for scores on the progressive matrices

Score	Description
5 (≥ 55)	Intellectually Superior
4 (45–54)	Above average intellectual capacity
3 (30–44)	Average intellectual capacity
2 (11–29)	Below average intellectual capacity
1 (≤ 10)	Intellectually inferior
The mean score was obtained for each grade and converted to the above scores and individual scores were compared with the converted mean score.	
